# Supplementary material for: Identification of photoperiod-induced specific miRNAs in the adrenal glands of Sunite sheep (Ovis aries)
Source: Front Vet Sci. 2022 Jul 22;9:888207. doi: 10.3389/fvets.2022.888207 (PMC9354845; doi:10.3389/fvets.2022.888207)
Supplement: Supplementary file 1 [file Data_Sheet_1.ZIP › Supplementary file/miRDeep2 file.zip!/miRDeep2 file/Know_Structure/SP42A3/oar-mir-493.pdf]

miRBase precursor : oar-mir-493
Total read count : 8583
oar-miR-493-5p read count : 6350
oar-miR-493-3p read count : 2233
remaining reads : 0

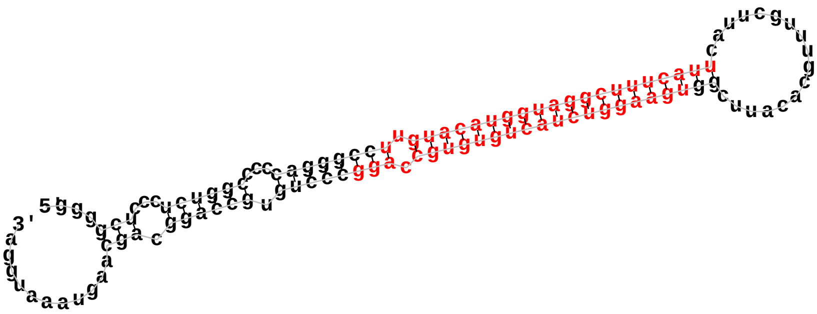

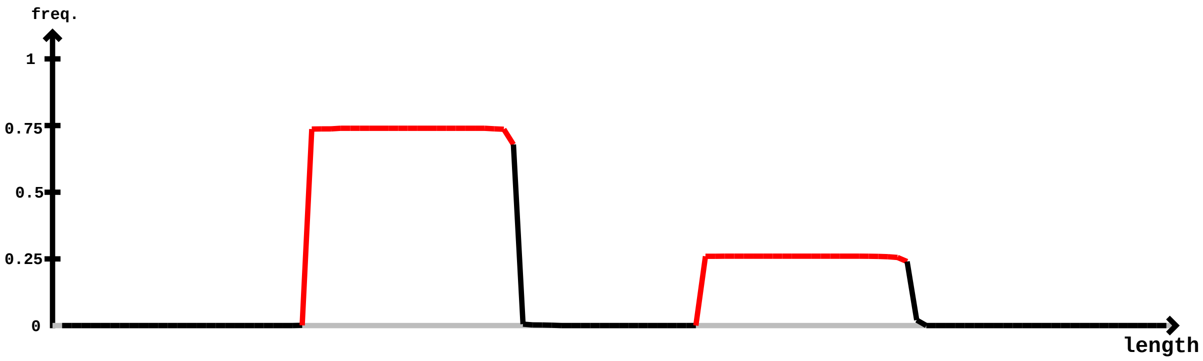

oar-miR-493-5p

oar-miR-493-3p

5'- ggggcucccucuggcccccagggccuuguacaugguaggcuuucauucauucguuugcacauucggugaaggucuacugugugccaggcccugugccaggcagcaaguaaaugga -3'	exp		
...(((...((((((...((((((((.((((((((((((((((((((..................))))))))))))))))))))).)))))))).)))))).))))..........	reads	mm	sample
........................cuuguacaugguaggcuuuca..	1	0	seq
........................cuuguacaugguaggcuuucauu..	7	0	seq
.........................uuguacaugguaggcuuuc...	18	0	seq
.........................uuguacaugguaggcuuuca..	13	0	seq
.........................uuguacaugguaggcuuucau...	489	0	seq
.........................uuguacaugguaggcuuucauu..	5772	0	seq
.........................uuguacaugguaggcuuucauuc...	23	0	seq
.........................uuguacaugguaggcuuucauuca..	1	0	seq
..........................uguacaugguaggcuuucauu..	2	0	seq
..........................uguacaugguaggcuuucauuc...	2	0	seq
..........................uguacaugguaggcuuucauuca..	1	0	seq
...........................guacaugguaggcuuucau...	1	0	seq
............................uacaugguaggcuuucauu..	1	0	seq
............................uacaugguaggcuuucauuca..	1	0	seq
............................uacaugguaggcuuucauucau...	7	0	seq
............................uacaugguaggcuuucauucauu..	11	0	seq
....gugaaggucuacugugugccagg...........................	1	0	seq
....ugaaggucuacugugug................................	2	0	seq
....ugaaggucuacugugugU...............................	2	1	seq
....ugaaggucuacugugugc...............................	2	0	seq
....ugaGggucuacugugugc...............................	1	1	seq
....ugaaggucuacugugugcc..............................	11	0	seq
....ugaaggucuacugugugcca.............................	23	0	seq
....ugaaggCcuacugugugccag............................	1	1	seq
....ugaaggucuacugugugccag............................	128	0	seq
....ugaaggucuGcugugugccag............................	2	1	seq
....ugaaUgucuacugugugccagg...........................	1	1	seq
....ugaaggucuacuAugugccagg...........................	5	1	seq
....ugaaggCcuacugugugccagg...........................	5	1	seq
....ugaaggucuGcugugugccagg...........................	4	1	seq
....ugaagAucuacugugugccagg...........................	1	1	seq
....ugaaggucuacugugugccagg...........................	1866	0	seq
....ugaaggucuacugugugccaggc..........................	175	0	seq
....aaggucuacugugugccagg...........................	1	0	seq

oar-miR-493-5p

oar-miR-493-3p

ggggcucccucuggcccccagggccuuguacaugguaggcuuucauucauucguuugcacauucggugaaggucuacugugugccaggcccugugccaggcagcaaguaaaugga

....aaggucuacugugugccaggc......................... 2 0 seq
